# All-*trans* Retinoic Acid Inhibits Hepatitis B Virus Replication by Downregulating HBx Levels via Siah-1-Mediated Proteasomal Degradation

**DOI:** 10.3390/v15071456

**Published:** 2023-06-27

**Authors:** Jiwoo Han, Kyung Lib Jang

**Affiliations:** 1 Department of Integrated Biological Science, The Graduate School, Pusan National University, Busan 46241, Republic of Korea; hanjiwoo@pusan.ac.kr; 2 Department of Microbiology, College of Natural Science, Pusan National University, Busan 46241, Republic of Korea; 3 Microbiological Resource Research Institute, Pusan National University, Busan 46241, Republic of Korea

**Keywords:** all-*trans* retinoic acid, HBx, HBV, p53, Siah-1, ubiquitin–proteasome system

## Abstract

All-*trans* retinoic acid (ATRA), the most biologically active metabolite of vitamin A, is known to abolish the potential of HBx to downregulate the levels of p14, p16, and p21 and to stimulate cell growth during hepatitis B virus (HBV) infection, contributing to its chemopreventive and therapeutic effects against HBV-associated hepatocellular carcinoma. Here, we demonstrated that ATRA antagonizes HBx to inhibit HBV replication. For this effect, ATRA individually or in combination with HBx upregulated p53 levels, resulting in upregulation of seven in absentia homolog 1 (Siah-1) levels. Siah-1, an E3 ligase, induces ubiquitination and proteasomal degradation of HBx in the presence of ATRA. The ability of ATRA to induce Siah-1-mediated HBx degradation and the subsequent inhibition of HBV replication was proven in an in vitro HBV replication model. The effects of ATRA became invalid when either p53 or Siah-1 was knocked down by a specific shRNA, providing direct evidence for the role of p53 and Siah-1 in the negative regulation of HBV replication by ATRA.

## 1. Introduction

Retinoids are natural or synthetic vitamin A derivatives and analogs implicated in several essential biological processes, including vision, development, differentiation, growth, metabolism, and cellular homeostasis [1]. All-*trans* retinoic acid (ATRA), the most biologically active metabolite of vitamin A, was initially approved for the treatment of acute promyelocytic leukemia (APL) [2] and has been increasingly included in both chemopreventive and therapeutic schemes for diverse human diseases, including cancer and skin disorders [3]. The pharmacological effects of ATRA are mainly attributed to the transcriptional regulation of associated genes by homo- and heterodimers of retinoid X and retinoic acid receptors (RARs) [4]. For example, patients with APL achieve complete remission via progressive differentiation of leukemic cells, as ATRA dissociates corepressors from promyelocytic leukemia protein (PML)-RARα oncoprotein, opens the chromatin structure, and recruits a coactivator that converts PML-RARα from a repressor to an activator [5]. Although the numbers of clinical trials and pharmaceutical applications of ATRA are steadily increasing, the detailed mechanisms underlying its pharmacological effects remain largely unknown.

Hepatitis B virus (HBV) is closely associated with the development of human liver diseases including hepatitis, liver cirrhosis, and hepatocellular carcinoma (HCC) [6,7]. As the representative of the family *Hepadnaviridae*, HBV contains a partially double-stranded circular DNA genome of approximately 3200 base pairs, which is prepared via reverse transcription of pre-genomic RNA [6,8]. Among the four open reading frames of HBV, S, C, P, and X, the shortest one encodes a 17-kDa multifunctional protein termed HBV X protein (HBx). HBx has been implicated in hepatocarcinogenesis because of its role in the regulation of cellular signal transduction pathways, transcriptional activation of cellular genes, and dysregulation of cell proliferation, apoptosis, and lipid metabolism [7,9]. In addition, the role of HBx as an activator of HBV replication has been demonstrated in numerous experimental models, including human hepatocyte chimeric mice [10], in vitro infection systems [11], and murine hydrodynamic injection [12]. HBx activates four viral promoters to produce HBV mRNA and pre-genomic RNA from a covalently closed circular DNA genome [6,13,14]. HBx also stimulates HBV replication indirectly by dysregulating cellular signal transduction pathways, such as the cytosolic calcium signaling pathway [15] and the phosphatidylinositol 3-kinase/Akt pathway [16]. Despite concrete evidence regarding the positive role of HBx in HBV replication, the mechanism of HBx regulation during HBV replication remains unclear.

Several studies have demonstrated that HBx and ATRA regulate the expression of genes involved in cell growth. For example, HBx stimulates cell growth by transcriptionally repressing the expression of p21 and p16 [17,18], whereas ATRA inhibits cell growth by upregulating the levels of p14, p16, and p21 [19,20]. It has been further demonstrated that HBx and ATRA antagonize each other in terms of the expression of p14, p16, and p21 and subsequent regulation of cell growth [21,22,23], which may contribute to their roles as positive and negative regulators of HCC progression, respectively. Despite numerous studies concerning antagonism between ATRA and HBx in the regulation of cell growth, the effects of ATRA on HBV replication, which can be considered another aspect of the interaction between HBV and ATRA, are completely unknown. This seems to be mainly attributable to difficulties in propagating HBV in cultured cells, which have been mostly resolved by recent advances in HBV infection and replication systems in vitro [24].

According to previous reports, HBx levels are primarily regulated by an E3 ligase, seven in absentia homolog 1 (Siah-1), which induces ubiquitination and proteasomal degradation of HBx [25,26]. In addition, p53 induces transcriptional activation of Siah-1 through p53 response elements located within its promoter [26]. Moreover, ATRA upregulates p53 by activating p14 expression via promoter hypomethylation [19], although the detailed mechanism remains unknown. These observations prompted us to investigate whether ATRA stimulates Siah-1 expression via p53 activation and subsequently inhibits HBV replication by lowering HBx levels via ubiquitin (Ub)-dependent proteasomal degradation. The present study employed an in vitro HBV replication system, which was recently optimized to support efficient HBV replication in cultured cells [27], thus providing an ideal experimental tool that enabled us to evaluate the effects of ATRA on HBV replication. Initially, we examined whether ATRA inhibited HBV replication via p53 activation. Secondly, we explored whether HBx was responsible for the p53-dependent inhibition of HBV replication by ATRA_._ Thirdly, we examined whether ATRA downregulated HBx levels via Siah-1-mediated proteasomal degradation. Finally, we sought to demonstrate that ATRA inhibits HBV replication by lowering HBx levels via Siah-1-mediated ubiquitination and proteasomal degradation in a p53-dependent manner.

## 2. Materials and Methods

### 2.1. Plasmids

The plasmid pCMV-3 × HA1-HBx (HA-HBx), which encodes HBx (genotype D) downstream of influenza virus haemagglutinin (HA) tag (YPYDVPDYA), was previously described [17]. The 1.2-mer wild-type (WT) HBV replicon, which contains 1.2 units of the HBV genome (1.2-mer WT; genotype D), and its HBx-null counterpart (1.2-mer HBx-null) were previously described [28]. The HBV core promoter/enhancer reporter, pHBV-luc, was previously described [28]. The plasmid pSiah-1-Myc WT encoding Myc-tagged Siah-1 was previously described [29]. The plasmid RC210241 (Cat No. 003049) encoding human sodium taurocholate cotransporting polypeptide (NTCP) was purchased from OriGene (Rockville, MD, USA). Scrambled (SC) short hairpin RNA (shRNA) (Cat No. sc-37007) and p53 shRNA (Cat. sc-29435) were obtained from Santa Cruz Biotechnology (Santa Cruz, CA, USA). Siah-1 shRNA (Cat No. SHCLND-NM003031) was obtained from Sigma-Aldrich (St. Louis, MO, USA). The plasmid pCH110 (Cat No. 27-4508-01) encoding the *Escherichia coli* β-galactosidase (*β-Gal*) gene was purchased from Addgene (Watertown, MA, USA). pCMV p53-WT and pHA-Ub were kindly provided by Dr. C.-W. Lee (Sungkyunkwan University, Suwon, Republic of Korea) and Dr. Y. Xiong (UNC-Chapel Hill, NC, USA), respectively.

### 2.2. Cell Culture and Transfection

HepG2 (Cat No. 88065) and Hep3B (Cat No. 88064) cell lines were obtained from the Korean Cell Line Bank (KCLB, Seoul, Republic of Korea). For transient expression, 2 × 10^5^ cells per well in a 6-well plate were transfected using TurboFect transfection reagent (Thermo Fisher Scientific, Waltham, MA, USA, Cat No. R0532) with the indicated amounts of plasmids and an empty vector supplemented to make the final amount of cocktails equivalent. Two NTCP-expressing cell lines, HepG2-NTCP and Hep3B-NTCP, were obtained by transfection with RC210241, followed by selection with 500 μg·mL^−1^ G418 sulfate (Sigma-Aldrich, Cat No. A1720). All cells were cultured in Dulbecco’s modified Eagle’s medium (DMEM) (WelGENE, Gyeongsan, Republic of Korea, Cat No. LM001-05) supplemented with 10% fetal bovine serum (Capricorn Scientific, Ebsdorfergrund, Germany, Cat No. FBS-22A), 100 units·mL^−1^ penicillin G (Sigma-Aldrich, Cat P3032-25MU), and 100 μg·mL^−1^ streptomycin (United States Biological, Salem, MA, USA, Cat No. 21865) at 37 °C in 5% CO_2_ in a humidified atmosphere. Cells were treated with ATRA (Sigma-Aldrich, Cat No. R2625), pifithrin-alpha (PFT-α) (Sigma-Aldrich, Cat No. P4359), cycloheximide (CHX) (Sigma-Aldrich, Cat No. C7698), or MG132 (Millipore, Burlington, MA, USA, Cat No. 474790) as necessary, under the indicated conditions.

### 2.3. Luciferase Reporter-Gene Assay

Approximately 1 × 10^5^ cells per well in 12-well plates were transfected with 0.3 µg of pHBV-luc along with the indicated plasmids. To control for transfection efficiency, 0.1 µg pCH110 was co-transfected as an internal control. Forty-eight hours after transfection, the luciferase assay was conducted using the Luciferase Reporter 1000 Assay System (Promega, Madison, WI, USA, Cat No. E4550). Luciferase activity measured using a microplate luminometer (LuBi MicroDigital, Seongnam, Republic of Korea) was normalized to β-gal activity, which was measured in corresponding cell extracts using O-nitrophenyl-β-D-galactopyranoside (Thermo Fisher Scientific, Cat No. 34055).

### 2.4. HBV Cell Culture System

For HBV seed preparation, Hep3B-NTCP cells were transfected with the 1.2-mer HBV replicon plasmids, as described above. The virus particles in the culture supernatant were amplified for HBV stock preparation by infecting Hep3B-NTCP cells for 4 days, as described below. The virus titer in HBV stock was measured using quantitative real-time polymerase chain reaction (qPCR), as described in the next section. HBV infection was performed in 6-well plates at a multiplicity of infection (MOI) of 50 for 4 days, according to a previously optimized HBV cell culture system with slight modifications [30]. Briefly, 2 × 10^5^ cells were inoculated with 1 × 10^7^ genome equivalents (GEQ) of HBV particles and incubated for 24 h in serum-free DMEM, which contained 4% polyethylene glycol 8000 (PEG 8000; Sigma-Aldrich, Cat No. D4463) and 2% dimethyl sulfoxide (DMSO; Sigma-Aldrich, Cat No. D8418). After rinsing twice with DMEM, the cells were cultured in DMEM supplemented with 3% FBS, 4% PEG 8000, and 2% DMSO for an additional 3 days.

### 2.5. Quantitative Real-Time PCR of HBV DNA

The HBV titer in the culture supernatant was measured using qPCR as previously described [31]. Briefly, HBV genomic DNA was purified from the culture supernatant using the QIAamp DNA Mini Kit (Qiagen, Hilden, Germany, Cat No. 51306). For conventional PCR analysis, the purified HBV genomic DNA was amplified using 2 × Τaq PCR Master Mix 1 (BioFACT, Daejeon, Republic of Korea, Cat No. ST301-19h) and a primer pair, HBV 1399F (5′-TGGTACCTGCGCGGGACGTCCTT-3′) and HBV 1632R (5′-AGCTAGCGTTCACGGTGGTCTCC-3′). For qPCR analysis, HBV DNA was amplified with SYBR Premix Ex Taq II (Takara Bio, Kusatsu, Japan, Cat No. RR82LR), HBV 379F (5′-GTGTCTGCGGCGTTTTATCA-3′), and HBV 476R (5′-GACAAACGGGCAACATACCTT-3′) using a Rotor Gene qPCR instrument (Qiagen).

### 2.6. Immunoprecipitation

The immunoprecipitation (IP) assay was conducted with the Classic Magnetic IP/Co-IP Kit (Thermo Fisher Scientific, Cat No. 88804). Briefly, 4 × 10^5^ cells per 60 mm plate were either transiently transfected with the designated plasmids or infected with HBV under the indicated conditions. Cell lysates were incubated with an anti-HBx antibody (Millipore, Cat No. 8419) overnight at 4 °C to allow for the formation of immune complexes. After washing, the immune complexes were harvested by incubation with Protein A/G magnetic beads. The beads were then collected using a magnetic stand (Pierce, Waltham, MA, USA) to obtain the antigen/antibody complexes, which were subjected to western blotting using the indicated antibodies.

### 2.7. Western Blot Analysis

For cell lysate preparation, cells were lysed in buffer (50 mM Tris-HCl (pH 8.0), 150 mM NaCl, 0.1% SDS, and 1% NP-40) supplemented with protease inhibitors (Roche, Basel, Switzerland, Cat No. 11836153001). The protein concentration was measured using a protein assay kit (Bio-Rad, Hercules, CA, USA, Cat No. 5000006). Proteins separated by SDS-PAGE were transferred onto a nitrocellulose blotting membrane (Amersham Bioscience, Amersham, UK, Cat No. 10600003) and incubated with primary antibodies against Siah-1 (Abcam, Cambridge, UK, Cat No. ab2237, 1:2000 dilution), p53 upregulated modulator of apoptosis (PUMA) (Cell Signaling Technology, Danvers, MA, USA, Cat No. 49765, 1:1000 dilution), HBx (Millipore, Cat No. MAB8419, 1:500 dilution), p53 (Santa Cruz Biotechnology, Cat No. sc-126, 1:1000 dilution), HA (Santa Cruz Biotechnology, Cat No. sc-7392, 1:500 dilution), HBV core antigen (HBcAg) (Santa Cruz Biotechnology, Cat No. sc-52400, 1:300 dilution), HBV surface antigen (HBsAg) (Santa Cruz Biotechnology, Cat. sc-53300, 1:400 dilution), Siah-1 (97-298) (Santa Cruz Biotechnology, Cat. sc-81785, 1:200 dilution), and γ-tubulin (Santa Cruz Biotechnology, Cat. No. sc-17787, 1:500 dilution), followed by subsequent incubation with an appropriate HRP-conjugated anti-mouse secondary antibody (Bio-Rad, Cat No. BR170-6516, 1:3000 dilution), anti-rabbit IgG (H+L)-HRP (Bio-Rad, Cat No. BR170-6515, 1:3000 dilution) or anti-goat IgG (H+L)-HRP (Thermo Scientific Scientific, Cat No. 31400, 1:10,000 dilution). An ECL kit (Advansta, San Jose, CA, USA, Cat No. K-12043-D20) was used to visualize the protein bands on the membrane using the ChemiDoc XRS imaging system (Bio-Rad).

### 2.8. Cell Viability Analysis

For determination of viable cells, an MTT assay was performed as previously described [32]. Briefly, cells were seeded at 1 × 10^4^ cells per well in 96-well plates and incubated under the indicated conditions. The cells were then treated with 10 µM 3-(4,5-dimethylthiazol-2-yl)-2,5-diphenyltetrazolium bromide (MTT, Affymetrix, Cleveland, OH, USA, Cat. No. 19265) for 4 h at 37 °C. The formazan compounds derived from the reduction of MTT by the mitochondrial dehydrogenases of the living cells were then dissolved in DMSO, and quantified by measuring the absorbance at 550 nm.

### 2.9. Statistical Analysis

Values indicate means ± standard deviation from at least three different experiments. Two-tailed Student’s *t*-tests were conducted for all statistical analyses. A *p*-value > 0.05 was considered statistically non-significant, whereas a *p*-value ≤ 0.05 was statistically significant.

## 3. Results

### 3.1. ATRA Inhibits HBV Replication in A p53-Dependent Manner

**Figure 1:**
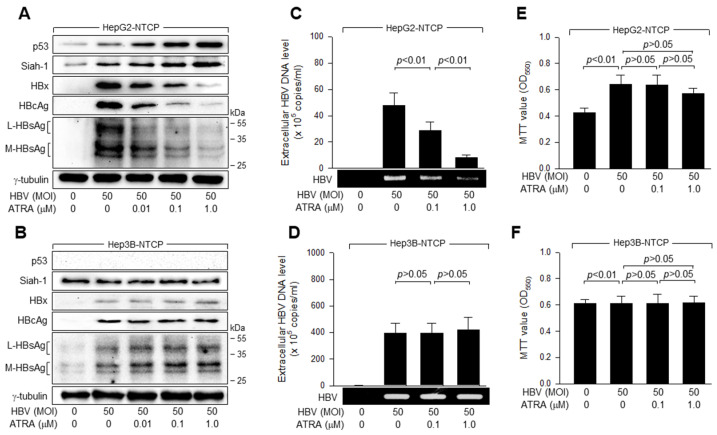
ATRA inhibits HBV replication in a p53-dependent manner. HepG2-NTCP and Hep3B-NTCP cells were infected with HBV at the indicated MOI for 24 h, washed twice with serum-free DMEM, and then incubated for an additional 3 days in DMEM containing 3% FBS, 4% PEG 8000, and 2% DMSO. Cells were either mock-treated or treated with the indicated concentrations of ATRA for 24 h before harvesting. (**A**,**B**) Cell lysates were subjected to western blotting to measure levels of p53, Siah-1, HBx, HBcAg, HBsAg, and γ-tubulin. (**C**,**D**) The levels of HBV particles released from the cells prepared in (**A**,**B**) were determined using conventional PCR and quantitative real-time PCR (qPCR). Results are shown as means ± standard deviation (SD) obtained from four independent experiments (*n* = 4). (**E**,**F**) Cell viability was measured using the MTT assay (*n* = 9).

Initially, we investigated whether ATRA differentially affected HBV replication in human hepatoma cells, depending on the status of p53. In this study, two human hepatoma cell lines, HepG2 and Hep3B, were used as experimental models. These cell lines are commonly employed in hepatology and liver cancer research due to their relevance to hepatocyte biology and their ability to support the replication of HBV. In particular, HepG2 cells but not Hep3B cells express WT p53, providing a unique platform for parallel comparisons of the roles of p53 in HBV-related molecular mechanisms [33]. HepG2-NTCP and Hep3B-NTCP, stably expressing the HBV receptor NTCP [34,35], were infected with HBV particles obtained from a 1.2-mer HBV replicon [28]. Replication of HBV in these cells was confirmed by western blotting analysis of viral proteins, including HBx, HBcAg, and HBsAg, in cell lysates (Figure 1A,B), and by measurement of virus particles in the culture medium using conventional PCR and qPCR (Figure 1C,D). Two different HBsAg, namely large (L)- and middle (M)-HBsAg, were detected in infected cells (Figure 1A,B), as previously demonstrated [31]. Unlike HepG2-NTCP cells, tiny amounts of HBsAg originating from the integrated HBV genome [36] were detected in the cell lysates of uninfected Hep3B-NTCP cells (Figure 1B, lane 1), whereas both conventional PCR and qPCR failed to detect evidence of HBV replication in these cells (Figure 1D). ATRA treatment downregulated the levels of intracellular HBV proteins and extracellular virus particles during HBV replication in HepG2-NTCP cells (Figure 1A,C), whereas these effects were negligible in Hep3B-NTCP cells in which p53 was absent (Figure 1B,D). The negative regulation of HBV replication by ATRA in HepG2 cells appeared to be specific, as treatment with ATRA at a concentration of 1.0 μM or lower did not result in significant side effects on MTT activity, which is commonly used as an indicator of cell viability, proliferation, and cytotoxicity (Figure 1E,F). Based on these observations, we concluded that ATRA inhibits HBV replication in human hepatoma cells in a p53-dependent manner.

### 3.2. ATRA Upregulates p53 Levels to Inhibit HBV Replication

Next, we investigated how ATRA inhibits HBV replication in a p53-dependent manner. Consistent with previous reports [26,37], HBV infection upregulated p53 levels in HepG2-NTCP cells and ATRA augmented this effect in a dose-dependent manner (Figure 1A). Accordingly, p53 levels were inversely proportional to HBV propagation rates, as represented by the levels of intracellular viral proteins and extracellular virus particles in the presence of ATRA (Figure 1A,C). These results were consistent with a previous report demonstrating that p53 acts as a negative regulator of HBV propagation [31]. Based on these observations, we hypothesized that ATRA upregulates p53 to inhibit HBV replication in human hepatoma cells.

**Figure 2:**
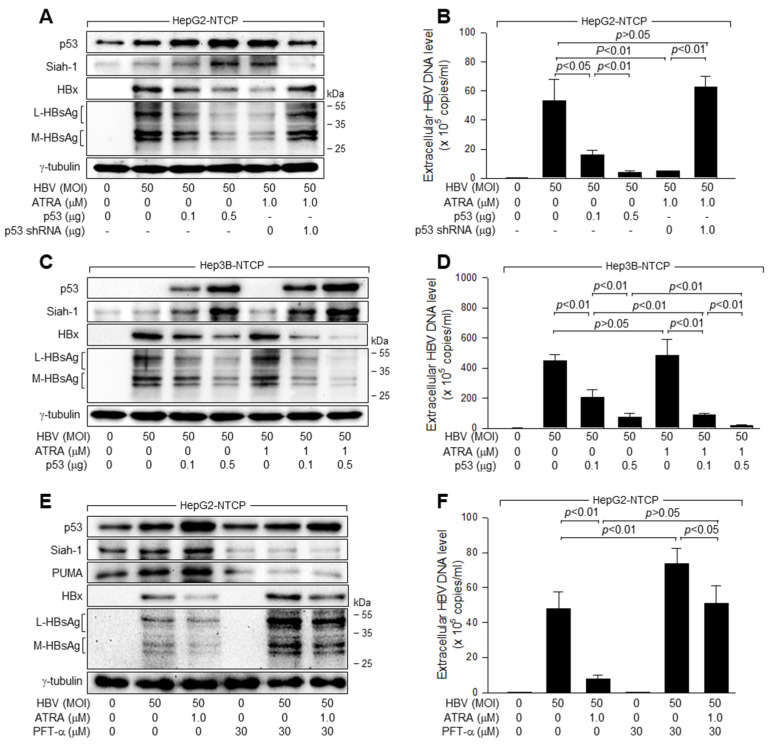
ATRA activates p53 to inhibit HBV replication in human hepatoma cells. (**A**) HepG2-NTCP cells were transiently transfected with the indicated amounts of p53, SC shRNA, and p53 shRNA plasmids for 24 h and then infected with HBV for 4 days as described in Figure 1, followed by western blotting analysis. For lanes 5 and 6, cells were treated with ATRA for 24 h before harvesting. (**B**) Levels of HBV particles in cell supernatants prepared in (**A**) were determined using qPCR (*n* = 3). (**C**) Hep3B-NTCP cells were transiently transfected with p53 expression plasmid for 24 h and then infected with HBV for 4 days as in Figure 1, followed by western blotting analysis. For lanes 5 to 7, cells were treated with ATRA for 24 h before harvesting. (**D**) Levels of HBV particles in cell supernatants prepared in (**C**) were determined using qPCR (*n* = 3). (**E**) HepG2-NTCP cells were transfected with p53 expression plasmid for 24 h and infected with HBV for an additional 4 days in the presence or absence of pifithrin-alpha (PFT-α), followed by western blotting analysis. For lanes 3 and 6, cells were treated with ATRA for 24 h before harvesting. (**F**) Levels of HBV particles in culture supernatants prepared in (**E**) were measured using qPCR (*n* = 4).

To confirm that ATRA inhibits HBV replication by upregulating p53 levels, we knocked down p53 in HepG2-NTCP cells and expressed ectopic p53 in Hep3B-NTCP cells before HBV infection and ATRA treatment. p53 knockdown almost completely abolished the ability of ATRA to inhibit HBV replication in HepG2-NTCP cells (Figure 2A,B), whereas ectopic p53 expression enabled ATRA to inhibit HBV replication in Hep3B- NTCP cells, in which ATRA upregulated exogenous p53 levels (Figure 2C,D). Consistent with a previous report [31], ectopic p53 expression without ATRA treatment was sufficient to inhibit HBV replication in both HepG2-NTCP and Hep3B-NTCP cells (Figure 2A–D). Taken together, we concluded that ATRA inhibits HBV replication via the upregulation of p53 levels in human hepatoma cells.

Activation of p53 by HBV infection resulted in the upregulation of two p53 targets, Siah-1 and PUMA, in HepG2-NTCP cells (Figure 2E). Treatment with ATRA further elevated protein levels by upregulating p53 levels in HBV-infected HepG2-NTCP cells (Figure 2E). To investigate whether p53 transcriptional activity is essential for ATRA-induced inhibition of HBV replication, we used a specific p53 inhibitor, PFT-α, which is known to prevent the transactivation of p53-responsive genes [38]. Treatment with PFT-α led to a dramatic decrease in the levels of Siah-1 and PUMA in HepG2-NTCP cells, irrespective of whether they were infected with HBV, and thus almost abolished the effect of HBV infection on Siah-1 and PUMA levels in these cells (Figure 2E). Additionally, PFT-α almost abolished the ability of ATRA to elevate Siah-1 and PUMA levels during HBV infection of HepG2-NTCP cells (Figure 2E). These results indicated that PFT-α effectively inhibited p53 transcriptional activity under our experimental conditions. As demonstrated by the elevated levels of intracellular HBV proteins and extracellular virus particles, PFT-α treatment resulted in an increase in HBV replication in HepG2-NTCP cells, both in the presence and absence of ATRA, thus abolishing the ability of ATRA to inhibit HBV replication (Figure 2E,F). These results indicated that p53 transcriptional activity is critical for the inhibition of HBV replication by ATRA in human hepatoma cells.

### 3.3. ATRA Lowers HBx Levels to Inhibit HBV Replication in a p53-Dependent Manner

**Figure 3:**
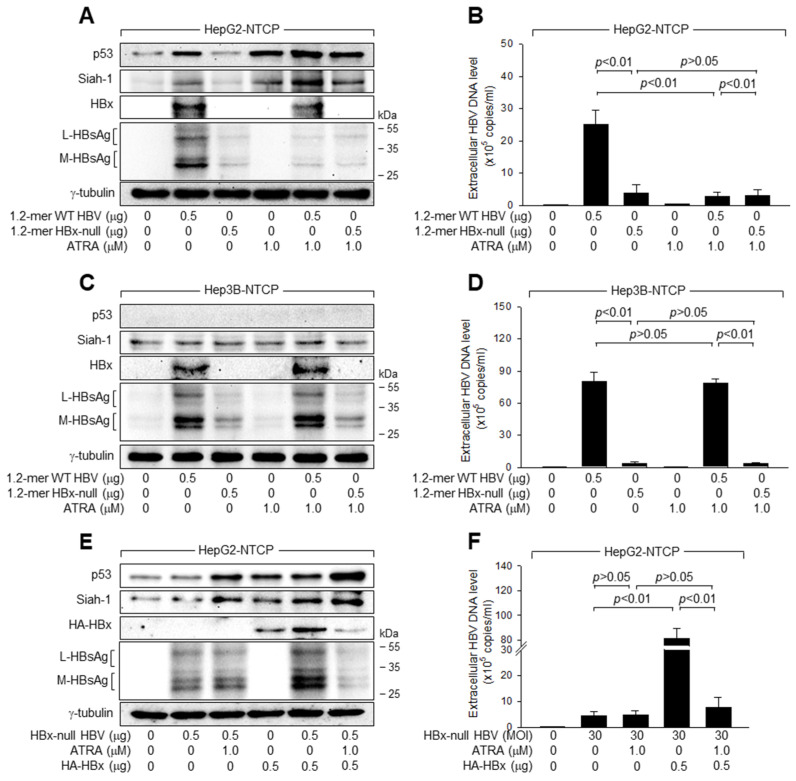
ATRA downregulates HBx levels to inhibit HBV replication in human hepatoma cells. HepG2-NTCP and Hep3B-NTCP cells were transiently transfected with a 1.2-mer WT HBV replicon (1.2-mer WT) or its HBx-null counterpart (1.2-mer HBx-null) with or without an HBx expression plasmid for 24 h and then treated with the indicated concentrations of ATRA for an additional 24 h. (**A**,**C**,**E**) Levels of the indicated proteins were measured by western blotting analysis. (**B**,**D**,**F**) Levels of extracellular HBV DNA were determined using qPCR (*n* = 3).

According to data obtained from an in vitro HBV infection system, ATRA downregulated the levels of HBx, an activator of HBV replication [11], in HepG2-NTCP cells but not in Hep3B-NTCP cells (Figure 1A,B), suggesting a possible role of HBx in the p53-dependent inhibition of HBV replication by ATRA in human hepatoma cells. To prove this hypothesis, 1.2-mer WT and 1.2-mer HBx-null [39] were employed. Transient transfection of HepG2-NTCP cells with 1.2-mer WT resulted in the upregulation of p53 and Siah-1 levels (Figure 3A), production of intracellular HBV proteins (Figure 3A), and secretion of extracellular virus particles (Figure 3B). In addition, treatment with ATRA upregulated p53 and Siah-1 levels in HepG2-NTCP cells, and this effect was greater in cells transfected with 1.2-mer WT (Figure 3A). Moreover, treatment with ATRA inhibited the replication of HBV derived from 1.2-mer WT in HepG2-NTCP cells, as proven by lowered levels of intracellular viral proteins and extracellular HBV particles derived from these cells (Figure 3A,B). However, these effects were negligible in Hep3B-NTCP cells (Figure 3C,D). These results indicated that p53-dependent inhibition of HBV replication by ATRA can be reproduced in a 1.2-mer HBV replicon system.

Transfection of HepG2-NTCP and Hep3B-NTCP cells with 1.2-mer HBx-null led to lower levels of intracellular HBV proteins and extracellular virus particles than those obtained with 1.2-mer WT (Figure 3A–D), suggesting that HBx is a positive regulator of HBV replication, irrespective of the presence of p53. In addition, unlike the 1.2-mer WT, 1.2-mer HBx-null failed to upregulate p53 levels in HepG2-NTCP cells, confirming the role of HBx as a potent inducer of p53 activation [26]. Moreover, ATRA was unable to inhibit the replication of HBx-null HBV in HepG2-NTCP and Hep3B-NTCP cells (Figure 3A–D). Accordingly, ATRA abolished the potential of HBx to stimulate HBV replication in HepG2-NTCP cells, whereas ATRA failed to induce this effect in Hep3B-NTCP cells, suggesting that HBx plays a major role in the p53-dependent inhibition of HBV replication by ATRA in human hepatoma cells. The importance of HBx was further investigated by ectopic HBx expression in HepG2-NTCP cells, which successfully complemented the defects of 1.2-mer HBx-null in terms of both p53 activation and HBV replication (Figure 3E,F). Ectopic HBx expression also enabled ATRA to inhibit the propagation of HBV derived from the 1.2-mer HBx-null in HepG2-NTCP cells (Figure 3E,F). Therefore, we concluded that ATRA inhibits HBV replication by lowering HBx levels in a p53-dependent manner.

### 3.4. ATRA Downregulates HBx Levels via Activation of p53 in Human Hepatoma Cells

**Figure 4:**
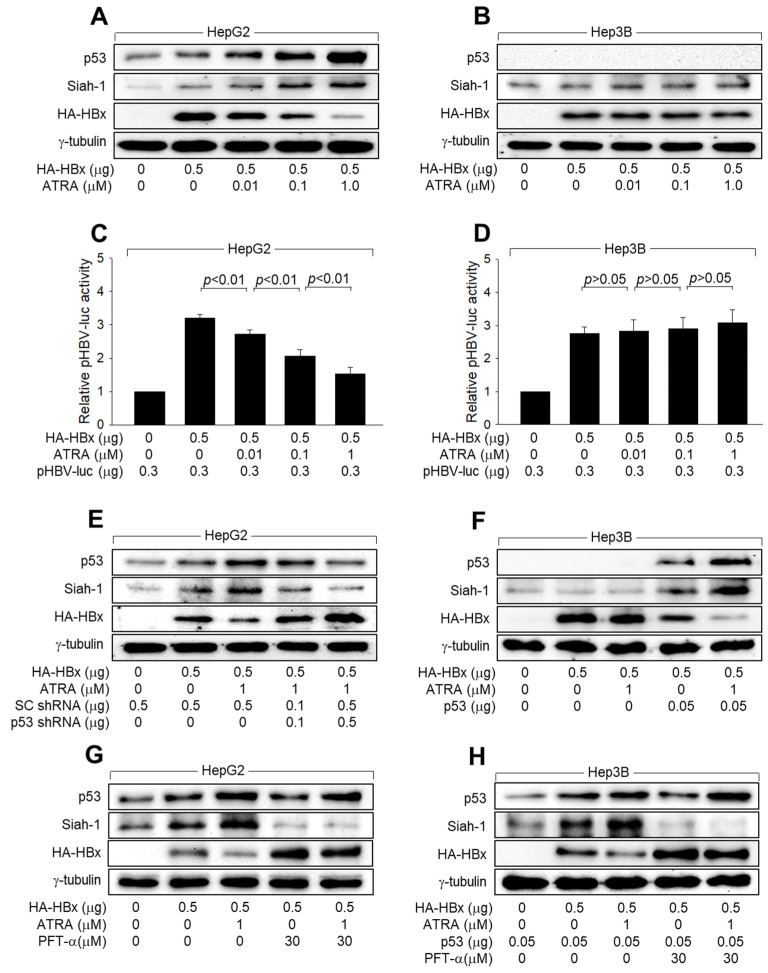
ATRA activates p53 to downregulate HBx levels in human hepatoma cells. (**A**,**B**,**E**–**H**) HepG2 and Hep3B cells were transiently transfected with HBx expression plasmid along with SC shRNA, p53 shRNA, or p53 expression plasmid for 24 h and either mock-treated or treated with ATRA for an additional 24 h, followed by western blotting analysis. For (**G**,**H**), cells were incubated in the presence or absence of PFT-α. (**C**,**D**) HepG2 and Hep3B cells were transfected with HBx expression plasmid and pHBV-luc, which contains the HBV core promoter [28], for 24 h and then treated with the indicated amounts of ATRA for an additional 24 h, followed by the luciferase assay. Values indicate relative luciferase activity compared to the basal levels of the control (*n* = 3).

Downregulation of HBx levels during HBV infection could be either a cause or result of ATRA-induced inhibition of HBV replication. This was confirmed by verifying whether ATRA downregulates ectopic HBx levels in the absence of HBV replication. Indeed, treatment with ATRA downregulated the levels of ectopically expressed HBx in HepG2 cells in a dose-dependent manner, and ATRA upregulated p53 levels (Figure 4A). ATRA inhibited HBx-mediated activation of the HBV core promoter in HepG2 cells, as demonstrated by reduced reporter activity from pHBV-luc containing the full-length HBV core promoter/enhancer in these cells (Figure 4C). In contrast, treatment with ATRA failed to induce any detectable changes in either HBx levels or transcriptional activity in Hep3B cells (Figure 4B,D).

To confirm the role of p53 in ATRA-induced HBx downregulation in human hepatoma cells, we knocked down p53 and HBx expression in ATRA-treated HepG2 cells. p53 knockdown upregulated HBx levels in the presence of ATRA (Figure 4E). In addition, ATRA upregulated exogenous p53 and downregulated HBx levels in Hep3B cells (Figure 4F). Moreover, ectopic p53 expression in the absence of ATRA was sufficient to downregulate HBx expression in Hep3B cells (Figure 4F). Taken together, we concluded that ATRA upregulates p53 to downregulate HBx during HBV infection in human hepatoma cells.

To investigate whether p53 transcriptional activity is required for ATRA-induced downregulation of HBx levels, we examined the effect of ATRA on HBx levels in the presence of PFT-α. Treatment with PFT-α drastically decreased Siah-1 levels in HepG2 and Hep3B cells expressing p53, irrespective of whether they were treated with ATRA or not (Figure 4G,H), indicating that PFT-α effectively inhibits p53 transcriptional activity under these conditions. Additionally, PFT-α dramatically upregulated HBx levels in the presence and absence of ATRA, abolishing the ability of ATRA to downregulate HBx levels in HepG2 and Hep3B cells expressing p53 (Figure 4G,H). These results indicated that p53 transcriptional activity is strongly implicated in the ATRA-induced downregulation of HBx levels in human hepatoma cells.

### 3.5. ATRA Activates Siah-1 Expression via Upregulation of p53 Levels to Downregulate HBx Levels

According to data obtained from the in vitro HBV infection and 1.2-mer HBV replicon systems, HBV infection upregulated the levels of Siah-1, an E3 ligase that mediates the ubiquitination and proteasomal degradation of HBx [25], in HepG2-NTCP cells but not in Hep3B-NTCP cells (Figure 1 and Figure 3). Ectopic HBx expression without the involvement of other viral proteins was sufficient to upregulate Siah-1 levels in HepG2 cells, but not in Hep3B cells (Figure 4). ATRA treatment further elevated Siah-1 levels in both HBV-infected HepG2-NTCP cells and HBx-expressing HepG2 cells (Figure 1A, Figure 3A and Figure 4A). Considering the role of p53 as a transcriptional activator of Siah-1 expression [40] and the potential of HBx and ATRA to upregulate p53 levels (Figure 1, Figure 2, Figure 3 and Figure 4), it can be assumed that ATRA and HBx, individually or in combination, activate Siah-1 expression via upregulation of p53 levels in human hepatoma cells. The role of p53 in the activation of Siah-1 expression by HBx and ATRA was confirmed by p53 knock-down in HepG2 cells and p53 overexpression in Hep3B cells, which resulted in decreased and increased Siah-1 levels, respectively (Figure 4E,F). Moreover, treatment with PFT-α almost abolished the ability of HBx and ATRA to lower Siah-1 levels (Figure 4G,H), indicating that p53 functions as a transcriptional activator of Siah-1 expression.

**Figure 5:**
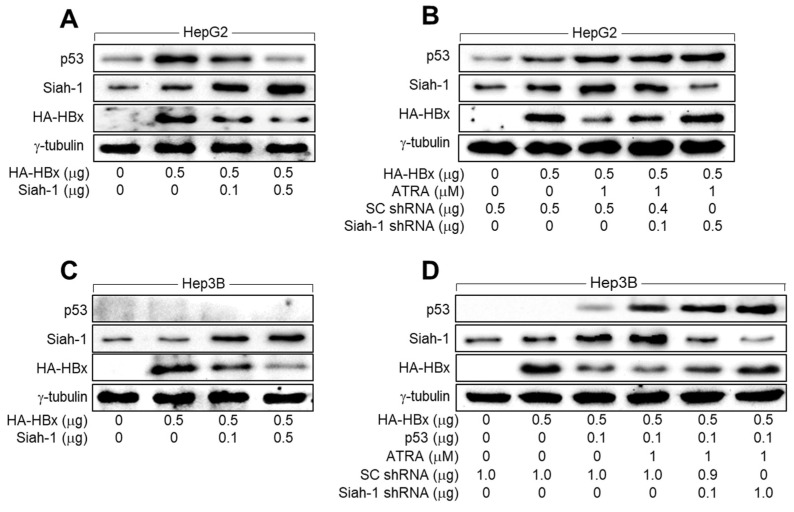
ATRA activates Siah-1 expression via activation of p53 to downregulate HBx levels. (**A**,**B**) HepG2 cells were transiently transfected with HBx expression plasmid along with Siah-1, SC shRNA, or Siah-1 shRNA for 24 h and treated with ATRA for an additional 24 h, followed by western blotting analysis. (**C**,**D**) Hep3B cells were transiently transfected with HBx expression plasmid along with Siah-1, SC shRNA, or Siah-1 shRNA for 24 h and treated with ATRA for an additional 24 h, followed by western blotting analysis.

We further investigated whether Siah-1 was involved in the p53-dependent downregulation of HBx levels by ATRA in human hepatoma cells. Data obtained from both HBV infection and HBx overexpression systems invariably exhibited an inverse correlation between Siah-1 and HBx levels in human hepatoma cells, irrespective of whether they were treated with ATRA (Figure 1, Figure 2, Figure 3 and Figure 4). In addition, ectopic Siah-1 expression lowered HBx levels in HepG2 and Hep3B cells, whereas Siah-1 knock-down elevated HBx levels in these cells (Figure 5), confirming that Siah-1 downregulated HBx levels via a mechanism that does not involve p53, as previously described [41]. Ectopic Siah-1 expression also downregulated p53 levels in HepG2 cells, whereas Siah-1 knockdown upregulated p53 levels in HepG2 and Hep3B cells (Figure 5), presumably because of the combination of the negative effect of Siah-1 on HBx levels and the positive effect of HBx on p53 levels. Taken together, we concluded that ATRA downregulates HBx levels by upregulating Siah-1 levels via p53 activation in human hepatoma cells.

### 3.6. ATRA Downregulates HBx Levels via Siah-1-Mediated Ubiquitination and Proteasomal Degradation in a p53-Dependent Manner

**Figure 6:**
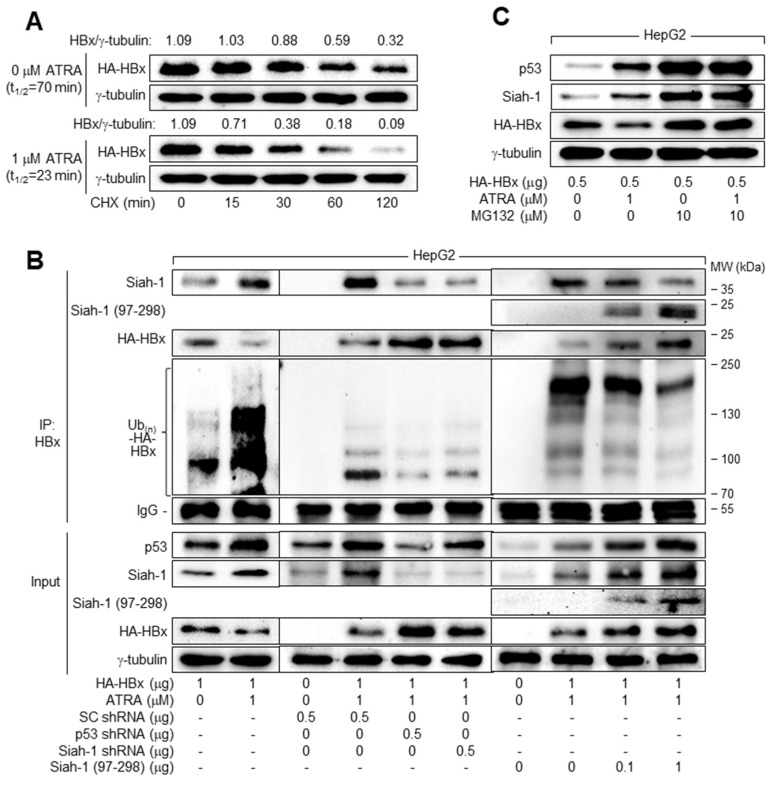
ATRA downregulates HBx levels via Siah-1-mediated ubiquitination and proteasomal degradation in a p53-dependent manner. (**A**) HepG2 cells transiently transfected with HBx for 48 h were treated with 50 μM cycloheximide (CHX) for the indicated time before harvesting to block further protein synthesis, followed by western blotting analysis. Levels of HBx and γ-tubulin were quantified using ImageJ 1.53k image analysis software (NIH) to reveal the levels of HBx relative to the loading control (γ-tubulin), which were used to determine HBx t_1/2_. (**B**) HepG2 cells were transiently transfected with either empty vector or HBx expression plasmid along with the indicated plasmids for 24 h and treated with ATRA for an additional 24 h. Total HBx protein in cell lysates was immunoprecipitated with an anti-HBx antibody and subjected to western blotting using anti-HBx, anti-Siah-1, and anti-HA antibodies to detect HBx, Siah-1, and HA-Ub-complexed HBx, respectively. The input indicated the levels of the indicated proteins in the cell lysates. (**C**) HepG2 cells transiently transfected with HBx expression plasmid and then treated with ATRA as above were treated with MG132 for 4 h before harvesting, followed by western blotting analysis.

Having established that ATRA downregulates HBx levels via upregulation of Siah-1 levels, we examined whether ATRA decreases the stability of HBx protein in human hepatoma cells in a p53-dependent manner. For this purpose, HepG2 cells expressing HBx were treated with CHX to block protein synthesis and HBx and γ-tubulin levels were measured in these cells (Figure 6A). The half-life (t_1/2_) of HBx was 70 min in HepG2 cells, which was drastically reduced by treatment with ATRA to 23 min, indicating that ATRA decreases HBx protein stability.

To further explore the role of Siah-1 in ATRA-induced downregulation of HBx levels, we examined whether ATRA increases Siah-1-mediated ubiquitination of HBx in a p53-dependent manner. For this purpose, we introduced HBx and HA-tagged Ub into HepG2 and Hep3B cells, with or without ATRA treatment, and immunoprecipitated Ub-complexed HBx. According to data from co-IP, Siah-1 interacted with HBx to induce its ubiquitination, as demonstrated by the smeared multiple bands of polyubiquitinated HBx in HepG2 cells (Figure 6B, lane 1). ATRA increased the interaction between HBx and Siah-1 in HepG2 cells, resulting in strong ubiquitination of HBx and subsequent downregulation of HBx protein levels (Figure 6B, lane 2). In addition, knockdown of p53 or Siah-1 in the presence of ATRA decreased the interaction between HBx and Siah-1 in HepG2 cells, resulting in a drastic decrease in the ubiquitination of HBx and subsequent upregulation of its protein levels (Figure 6B, lanes 5 and 6). The introduction of an N-terminal deletion mutant of Siah-1 (amino acids 97–298), which did not have the RING domain [29], also dramatically decreased the ubiquitination of HBx by competitively inhibiting the binding of endogenous Siah-1 to HBx, resulting in an increase in HBx protein levels and subsequent upregulation of p53 and Siah-1 levels (Figure 6B, lanes 9 and 10). Consistently, treatment with the universal proteasomal inhibitor MG132 nearly abolished the ability of ATRA to regulate p53, Siah-1, and HBx levels, thus equalizing the protein levels of HBx in the presence and absence of ATRA (Figure 6C), confirming that ATRA downregulates HBx levels via Siah-1-mediated proteasomal degradation.

### 3.7. ATRA Downregulates HBx Levels via Siah-1-Mediated Proteasomal Degradation to Inhibit HBV Replication in a p53-Dependent Manner

**Figure 7:**
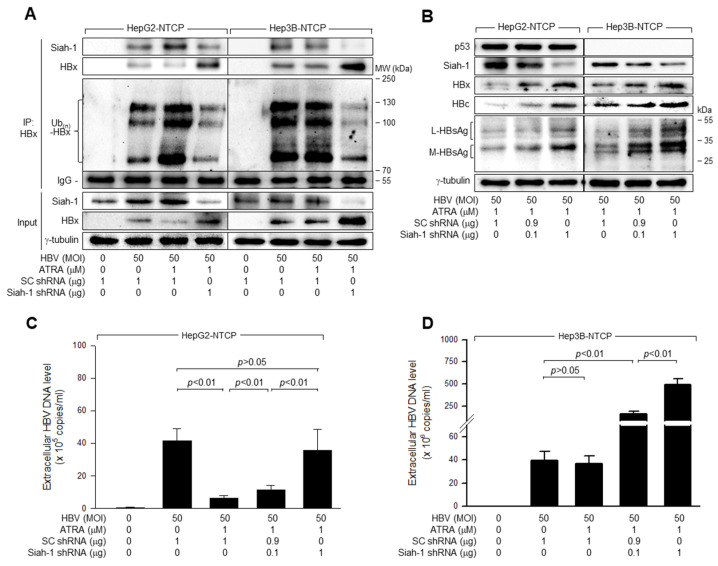
ATRA downregulates HBx levels via Siah-1-mediated proteasomal degradation to inhibit HBV replication in a p53-dependent manner. HepG2-NTCP and Hep3B-NTCP cells were transfected with the indicated amounts of SC shRNA and Siah-1 shRNA plasmids for 24 h and then either mock-infected or infected with HBV for 4 days. Cells were treated with ATRA at the indicated concentration for 24 h before harvesting. For (**A**), pHA-Ub was included in the transfection mixtures. (**A**) Total HBx protein was immunoprecipitated with an anti-HBx antibody as in Figure 6B and subjected to western blotting using anti-HBx, anti-Siah-1, and anti-HA antibodies to detect HBx, Siah-1, and HA-Ub-complexed HBx, respectively. The input indicated the levels of the indicated proteins in the cell lysates. (**B**) Levels of the indicated proteins were measured using western blotting analysis. (**C**,**D**) Levels of HBV particles in the culture supernatants were measured using qPCR (*n* = 4).

Consistent with data obtained from the HBx overexpression system (Figure 6B), treatment with ATRA increased the interaction between HBx and Siah-1 during HBV infection in HepG2-NTCP, resulting in strong ubiquitination of HBx and subsequent downregulation of its protein levels (Figure 7A, lane 3). In contrast, treatment with ATRA did not induce any detectable changes in the interaction between Siah-1 and HBx, HBx ubiquitination, or HBx protein levels during HBV infection of Hep3B-NTCP cells (Figure 7A, lane 7). In addition, knockdown of Siah-1 in the presence of ATRA decreased the interaction between HBx and Siah-1, resulting in a decrease in the ubiquitination of HBx and subsequent upregulation of its protein levels during HBV infection in both HepG2-NTCP and Hep3B-NTCP cells (Figure 7A, lanes 4 and 8), which indicated that Siah-1 can induce HBx ubiquitination during HBV infection via a p53-independent mechanism. Accordingly, Siah-1 knockdown stimulated HBV replication in the presence of ATRA in HepG2-NTCP and Hep3B-NTCP cells, as proven by an increase in the levels of intracellular HBV proteins and extracellular HBV particles (Figure 7B–D). Therefore, we concluded that ATRA inhibits HBV replication via Siah-1-mediated ubiquitination and proteasomal degradation of HBx in a p53-dependent manner.

## 4. Discussion

Retinoids are vitamin A derivatives and analogs that participate in a wide spectrum of biological processes from the embryo through adulthood [1]; thus, they have attracted considerable attention in diverse pharmaceutical research areas. The first successful application of retinoids in human disease was in APL, which is caused by a reciprocal chromosomal translocation between *RARα* and *PML* [2]. Thereafter, many findings on the roles of retinoids in the regulation of hematopoietic stem cells [42], tumor-initiating cells [43,44], immune cells [45], intestinal mucosa wound repair [46], cancer resistance [47], and cell reprogramming and differentiation [48] have further stimulated their pharmaceutical applications in cancer, skin pathologies, and other clinical conditions, such as inflammation, olfactory loss, and neuropsychiatric diseases [3]. The present study demonstrated that ATRA at pharmacological concentrations (0.01–1 μM), which has already been proven to induce the differentiation of APL cells [5], inhibits HBV replication in human hepatoma cells (Figure 1A,C), suggesting a possible application of ATRA in the development of an anti-viral drug against HBV. According to a previous report, treatment with ATRA for 24 h at 1 μM did not significantly affect the viability of HepG2 cells expressing HBx [22], which was also confirmed in the present study (Figure 1E,F). In addition, the present study demonstrated that the knockdown of Siah-1 using shRNA or the introduction of a dominant negative mutant of Siah-1 significantly attenuated the ability of ATRA to inhibit HBV replication (Figure 7B). This suggests that the observed anti-viral effect of ATRA is not attributed to its cytotoxicity but rather to its specific interaction with Siah-1.

Establishment of an efficient HBV replication system is an essential prerequisite for investigating the interactions between ATRA and HBV infection in vitro. HBV exhibits an extremely narrow host range. Several cell culture models have been established for HBV infection and replication. Primary human hepatocytes (PHHs) may exactly mimic HBV natural infection in vitro. However, several factors such as high cost, complex procedure, donor genetic variability, and rapid loss of susceptibility to HBV infection limit the application of PHH in HBV replication studies [49]. Similarly, HepaRG cells require a long differentiation process to obtain detectable levels of HBV replication [50]. Human HCC cell lines HepG2 and Huh7 can support HBV replication but not reinfection, primarily due to defects in the HBV receptor. The discovery of NTCP as the HBV receptor [35] made it possible to create HepG2, Huh7, and mouse hepatocyte line AML12 susceptible to HBV replication [51,52,53,54]. However, cell culture systems for HBV infection often require extremely high viral titers (1000–10,000 GEQ per cell) and fairly long incubation times (over 7 days) to obtain detectable levels of HBV infection [35,55]. Recently, optimizing infection conditions, for example by including DMSO and PEG 8000 in the culture media, made it possible to induce productive HBV infection with low starting inoculum concentrations (10–100 GEQ per cell) and short incubation periods (4–7 days) [30,53,56]. Consistently, our previous reports clearly detected several indicators of productive HBV infection, including intracellular proteins such as HBx and HBsAg, extracellular virions in the culture supernatant, and cccDNA in the Hirt extracts derived from HepG2-NTCP and Huh7D-NTCP cells infected with HBV at 30 MOI for 48 h [27,31]. The present study also provides several lines of evidence for HBV replication by detecting intracellular HBV proteins such as HBx, HBcAg, and HBsAg and extracellular virions from HepG2-NTCP and Hep3B-NTCP cells infected with HBV at 50 MOI for 4 days (Figure 1).

Previous reports demonstrated that HBx and ATRA oppositely regulate the expression of several tumor suppressor genes involved in cell growth control. For example, HBx inhibits the expression of p16 to stimulate cell growth and overcome stress-induced premature senescence and apoptosis [57]. In contrast, ATRA activates the expression of p14, p16, and p21 to induce cell growth arrest, senescence, and apoptosis [19,20,21]. It was further demonstrated that HBx and ATRA antagonize each other in the expression of p14, p16, and p21 and subsequent regulation of cell growth [22,23]. In the present study, we evidenced a new round of antagonism between ATRA and HBx in the regulation of HBV replication by demonstrating that HBx stimulates HBV replication (Figure 3E,F), as previously demonstrated [6,13,14], whereas ATRA inhibits HBV replication by lowering HBx levels in human hepatoma cells (Figure 1A,C). Interestingly, ATRA failed to inhibit the replication of HBx-null HBV (Figure 3A,B), whereas ectopic HBx expression was sufficient to inhibit the propagation of HBx-null HBV in HepG2-NTCP cells (Figure 3E,F), indicating that ATRA inhibits HBV replication primarily by downregulating HBx levels.

According to a previous report [19], ATRA upregulates p53 levels by activating the p14-MDM2 pathway, resulting in the transcriptional activation of p53 target genes such as *Bax* and *PUMA*. In addition, HBx is known to upregulate p53 levels by activating the ATM-Chk2 pathway, resulting in the activation of p53 target genes such as *p21*, proteasomal activator 28 gamma, *PUMA*, and *Siah-1* [17,22,26,37]. It has also been demonstrated that p53 decreases the protein stability of HBx in a Siah-1- and Ub-dependent manner, resulting in the inhibition of HBV replication [26,58]. Consistently, the present study provides several lines of evidence that support the hypothesis that ATRA inhibits HBV replication by lowering HBx levels via Siah-1-mediated proteasomal degradation. First, ATRA, individually or in combination with HBx, upregulated p53 and Siah-1 levels in both HBx overexpression and HBV infection systems (Figure 1, Figure 3 and Figure 4). Second, ATRA induced Siah-1-mediated polyubiquitination of HBx and decreased its stability (Figure 6). Third, ATRA inhibited HBV replication by upregulating Siah-1 and downregulating HBx levels (Figure 7). Fourth, all the effects of ATRA became invalid if Siah-1 was knocked down (Figure 5, Figure 6 and Figure 7), providing direct evidence for the role of Siah-1 in ATRA-mediated downregulation of HBx levels and subsequent inhibition of HBV replication. The effects of ATRA and HBx on p53 and Siah-1 appeared to be additive, as shown in Figure 3A. This suggests that both ATRA and HBx play roles, individually and in combination, in the inhibition of HBV replication mediated by ATRA. The findings suggest the existence of a negative feedback loop, where the presence of HBx contributes, at least in part, to the inhibitory effects of ATRA on HBV replication. The specific mechanisms underlying this feedback loop and the interactions between ATRA, HBx, p53, and Siah-1 require further investigation.

Interestingly, treatment with ATRA did not downregulate HBx levels and thus failed to inhibit HBV replication when p53 was inactivated by genetic defects (Figure 1B,D) or knocked down (Figure 2A,B and Figure 4E). In addition, ectopic p53 expression was sufficient to recover the ability of ATRA to lower HBx levels in Hep3B cells (Figure 2C,D and Figure 4F). Therefore, p53 appears to play a critical role in the ATRA-induced downregulation of HBx levels and inhibition of HBV replication. However, if ectopic Siah-1 expression downregulates HBx levels, whereas Siah-1 knockdown upregulates HBx levels, irrespective of the presence or absence of p53 (Figure 5, Figure 6 and Figure 7), p53 is not likely to directly participate in the Siah-1-mediated ubiquitination of HBx and may simply upregulate Siah-1 levels in response to ATRA. Treatment with PFT-α almost abolished the ability of ATRA to inhibit HBV replication (Figure 2E,F), confirming the importance of p53 transcriptional activity in this process. However, ATRA still significantly inhibited HBV replication in HepG2-NTCP cells in the presence of PFT-α (Figure 2E,F), suggesting that certain anti-HBV activities of ATRA may not require p53 transcriptional activity. Further studies are needed to clarify this issue. To the best of our knowledge, this is the first report demonstrating the negative effects of ATRA on HBV replication, suggesting that ATRA acts as a natural antiviral compound, protecting against HBV infection as a means of an innate host defense system. In addition, the present study provides a theoretical background that ATRA can be supplemented via medication and nutrition for therapeutic purposes against HBV infection in patients.

## Data Availability

The data presented in this study are available from the corresponding author upon reasonable request.
